# Prevalence of non-influenza respiratory viruses in acute respiratory infection cases in Mexico

**DOI:** 10.1371/journal.pone.0176298

**Published:** 2017-05-03

**Authors:** Larissa Fernandes-Matano, Irma Eloísa Monroy-Muñoz, Javier Angeles-Martínez, Brenda Sarquiz-Martinez, Iliana Donají Palomec-Nava, Hector Daniel Pardavé-Alejandre, Andrea Santos Coy-Arechavaleta, Clara Esperanza Santacruz-Tinoco, Joaquín González-Ibarra, Cesar Raúl González-Bonilla, José Esteban Muñoz-Medina

**Affiliations:** 1 Universidad del Valle de México Campus Chapultepec. Ciudad de México, México; 2 Laboratorio de Genómica, Departamento de Genética y Genómica Humana, Instituto Nacional de Perinatología “Isidro Espinosa de los Reyes”. Ciudad de México, México; 3 Laboratorio de Genómica, Departamento de Biología Molecular, Instituto Nacional de Cardiología “Ignacio Chávez”. Ciudad de México, México; 4 Laboratorio Central de Epidemiología, Centro Médico Nacional La Raza, Instituto Mexicano del Seguro Social. Ciudad de México, México; 5 División de Laboratorios de Vigilancia e Investigación Epidemiológica, Instituto Mexicano del Seguro Social. Ciudad de México, México; Kliniken der Stadt Köln gGmbH, GERMANY

## Abstract

**Background:**

Acute respiratory infections are the leading cause of morbidity and mortality worldwide. Although a viral aetiological agent is estimated to be involved in up to 80% of cases, the majority of these agents have never been specifically identified. Since 2009, diagnostic and surveillance efforts for influenza virus have been applied worldwide. However, insufficient epidemiological information is available for the many other respiratory viruses that can cause Acute respiratory infections.

**Methods:**

This study evaluated the presence of 14 non-influenza respiratory viruses in 872 pharyngeal exudate samples using RT-qPCR. All samples met the operational definition of a probable case of an influenza-like illness or severe acute respiratory infection and had a previous negative result for influenza by RT-qPCR.

**Results:**

The presence of at least one non-influenza virus was observed in 312 samples (35.8%). The most frequent viruses were rhinovirus (RV; 33.0%), human respiratory syncytial virus (HRSV; 30.8%) and human metapneumovirus (HMPV; 10.6%). A total of 56 cases of co-infection (17.9%) caused by 2, 3, or 4 viruses were identified. Approximately 62.5% of all positive cases were in children under 9 years of age.

**Conclusion:**

In this study, we identified 13 non-influenza respiratory viruses that could occur in any season of the year. This study provides evidence for the prevalence and seasonality of a wide range of respiratory viruses that circulate in Mexico and constitute a risk for the population. Additionally, our data suggest that including these tests more widely in the diagnostic algorithm for influenza may reduce the use of unnecessary antibiotics, reduce the hospitalisation time, and enrich national epidemiological data with respect to the infections caused by these viruses.

## Introduction

Acute respiratory infections (ARIs) represent the leading cause of morbidity and mortality worldwide [1–2], are the most common cause of outpatient care in adult patients [3], and are responsible for 70% of hospitalisations due to respiratory diseases in child populations aged 1–4 years and up to 90% in infants under 1 year of age [4].

ARIs are a group of diseases with normally less than 15 days of evolution that are caused by different microorganisms. A viral aetiological agent is estimated to be present in up to 80% of cases [5–7]. These infections can occur in the upper and lower respiratory tract. Upper ARIs may include one or more of the following conditions: rhinopharyngitis, pharyngoamygdalitis, sinusitis, and acute otitis media [8–9]. Lower ARIS include epiglottitis, laryngitis, laryngotracheobronchitis (croup), bronchitis, bronchiolitis, and pneumonia [9].

These infections are easily transmitted via coughing or sneezing. Contagion occurs through the inhalation of aerosols and microdroplets that contain the causative agent. Another important form of contagion is through direct contact of hands with objects contaminated with respiratory secretions from infected individuals, which can be self-inoculated into the nasal and buccal mucosae and/or into the ocular cavity [10].

A large amount of information is available concerning the timing and distribution of influenza viruses in the population following the reappearance of avian influenza A subtype H5N1 in 2003 and 2004 and the influenza A subtype H1N1 pandemic in 2009 [11]. Influenza viruses are one of the main causative agents of ARIs worldwide; however, many other respiratory viruses for which insufficient epidemiological information is available can also cause ARIs.

Studies performed at the international level have frequently identified human respiratory syncytial virus (HRSV), human parainfluenza virus (HPIV), influenza virus (flu), human mastadenovirus (HMdV), rhinovirus (RV), and enterovirus (EV) and less frequently identified human metapneumovirus (HMPV), primate bocaparvovirus (PBpV), and human coronavirus (HCoV) [12]. These viruses can serve as the causative agents for ARIs that occur outside of influenza season, when the rate of positivity can drop below 10%.

Although a large percentage of ARIs are caused by viral infections, the causative viruses have not been specifically identified in the majority of cases due to difficulties such as the high number of possible aetiological agents, similar symptoms among ARIs caused by different aetiological agents, the emergence of new viruses or new variants of previously described viruses, and the high cost of the detection tests [4]. Thus, little information is available concerning the prevalence and seasonality of these viruses, mainly in undeveloped countries, where the possibilities of carrying out this type of study on a regular basis is unusual.

The investigation of the causative agents of acute respiratory infections is important in order to lead the development of vaccines to target the most prevalent viruses and to reduce the unnecessary prescription of antibiotics and of antiviral oseltamivir phosphate. In addition, analysis like this can help to identify the age groups more susceptible to infection by each virus, in order to take the necessary actions for prevention and treatment.

Therefore, our aim was to determine the viral aetiology of ARIs in samples from patients who presented respiratory symptomatology but were negative for influenza by RT-qPCR testing.

## Methodology

### Study design

To evaluate the presence of noninfluenza respiratory viruses circulating in Mexican population in the different seasons of the year, we analized every pharyngeal exudate sample received by the Laboratorio Central de Epidemiología (LCE) (Central Epidemiology Laboratory) between the epidemiological week 40 of 2014 and the week 39 of 2015, according to the following criteria.

All samples had a previous negative result for influenza by RT-qPCR and met one of the following operational case definitions:

Influenza-like illness (ILI): A person of any age who presents a fever greater than or equal to 38°C, a cough, and cephalalgia accompanied by one or more of the following symptoms: rhinorrhoea, coryza, arthralgias, myalgias, prostration, odynophagia, thoracic pain, abdominal pain, or nasal congestion. In patients under five years of age, irritability is considered a cardinal sign in place of cephalalgia. In patients older than 65 years, fever is not required as a cardinal symptom.

Severe acute respiratory infection (SARI): A person of any age who presents difficulty breathing accompanied by a fever greater than or equal to 38°C and a cough with one or more of the following symptoms: poor general condition, thoracic pain, polypnoea or acute respiratory distress syndrome (ARDS) or any death associated with ILI or SARI.

A total of 872 samples were selected. (S1 Table)

### Total nucleic acid extraction

Total nucleic acids were obtained from a 200 μL pharyngeal exudate sample taken with a Dacron swab (Copan Diagnostics, Corona, Calif. Catalog: 159C) and stored in viral transport medium (BD™ Universal Viral Transport System, East Rutherford, New Jersey, EUA. Catalog: 220220) at -80°C prior to use. The MagNA Pure LC 2.0 Instrument (Roche Diagnostics, Mannheim, Germany) automated technology and the MagNA Pure LC Total Nucleic Acid Isolation Kit (Roche Diagnostics, Mannheim, Germany. Catalog: 03038505001) were used according to the manufacturer's instructions.

### RT-qPCR

The SuperScript™ III Platinum^®^ One-Step RT-qPCR System Kit (Invitrogen, Carlsbad, Califórnia, EUA. Catalog: 12574035) was used in a 7500 Fast Real-Time PCR System (Applied Biosystems^®^, Foster City, Califórnia, EUA) to amplify viral genetic material. Primers and probes were used for each of the following viruses: HMPV, HRSV, HPIV 1–4, betacoronavirus 1 (βCoV1), human coronavirus (HKU1, 229E, NL63) (HCoV), HMdV, RV, EV, and PBpV. The human RNAse P (RP) gene was used as an internal control (Table 1). The viruses were evaluated in uniplex reactions with the following reaction mixture: 12.5 μL of 2x Reaction Mix, 0.5 μL of each primer and probe, 1 μL of enzyme, 5.5 μL of RNAse-free water, and 5 μL of total nucleic acid. The following thermocycling conditions were used: one cycle of 45°C for 10 min and 95°C for 10 min and 45 cycles of 95°C for 15 sec and 55°C for 1 min.

**Table 1:** Sequence and working concentration of primers and probes.

Virus	Primer / Probe	Sequence	Conc μM
HMPV	PF	CAA GTG TGA CAT TGC TGA YCT RAA	30
	PR	ACT GCC GCA CAA CAT TTA GRA A	30
	Probe	FAM TGG CYG TYA GCT TCA GTC AAT TCA ACA GA BHQ-1	5
HRSV	PF	GGC AAA TAT GGA AAC ATA CGT GAA	25
	PR	TCT TTT TCT AGG ACA TTG TAY TGA ACA G	12.5
	Probe	FAM CTG TGT ATG TGG AGC CTT CGT GAA GCT BHQ-1	2.5
HPIV 1	PF	ACA AGT TGT CAA YGT CTT AAT TCR TAT	25
	PR	TCG GCA CCT AAG TAR TTY TGA GTT	25
	Probe	FAM ATA GGC CAA AGA T(BHQ-1) TG TTG TCG AGA CTA TTC CAA	2.5
HPIV 2	PF	GCA TTT CCA ATC TAC AGG ACT ATG A	37.5
	PR	ACC TCC TGG TAT AGC AGT GAC TGA AC	37.5
	Probe	FAM CCA TTT ACC T(BHQ-1) AA GTG ATG GAA TCA ATC GCA AA	2.5
HPIV 3	PF	TGG YTC AAT CTC AAC AAC AAG ATT TAA G	37.5
	PR	TAC CCG AGA AAT ATT ATT TTG CC	25
	Probe	FAM CCC RTC TGT(BHQ-1) TGG ACC AGG GAT ATA CTA CAA A	10
HPIV 4	PF	CTG CCA AAT CGG CAA TTA AAC	15
	PR	CTG GAC GCA ATC ATA AGR TGA TTC	15
	Probe	FAM CAT TAT TAT CTC TGC T(BHQ-1) TT CCT TAC AGG CCA CAT CA	5
HCoV 229E	PF	CAG TCA AAT GGG CTG ATG CA	37.5
	PR	AAA GGG CTA TAA AGA GAA TAA GGT ATT CT	25
	Probe	FAM CCC TGA CGA CCA CGT TGT GGT TCA BHQ-1	2.5
βCoV1	PF	CGA TGA GGC TAT TCC GAC TAG GT	25
	PR	CCT TCC TGA GCC TTC AAT ATA GTA ACC	37.5
	Probe	FAM TCC GCC TGG CAC GGT ACT CCC T BHQ-1	2.5
HCoV NL63	PF	GAC CAA AGC ACT GAA TAA CAT TTT CC	12.5
	PR	ACC TAA TAA GCC TCT TTC TCA ACC C	12.5
	Probe	FAM AAC ACG CTT(BHQ-1) CCA ACG AGG TTT CTT CAA CTG AG	2.5
HCoV HKU1	PF	CCT TGC GAA TGA ATG TGC T	5
	PR	TTG CAT CAC CAC TGC TAG TAC CAC	37.5
	Probe	FAM TGT GTG GCG GTT GCT ATT ATG TTA AGC CTG BHQ-1	2.5
HMdV	PF	GCC CCA GTG GTC TTA CAT GCA CAT C	25
	PR	GCC ACG GTG GGG TTT CTA AAC TT	25
	Probe	FAM TGC ACC AGA CCC GGG CTC AGG TAC TCC GA BHQ-1	5
RV	PF-1	CYA GCC TGC GTG GC	50
	PF-2	CYA GCC TGC GTG GT	50
	PR	GAA ACA CGG ACA CCC AAA GTA	50
	Probe	FAM TCC TCC GGC CCC TGA ATG YGG C BHQ-1	5
EV	PF	GGT GGC TGC GTT GGC	50
	PR	GAA ACA CGG ACA CCC AAA GTA	50
	Probe	FAM TCC TCC GGC CCC TGA ATG YGG C BHQ-1	5
PBpV	PF	TGC AGA CAA CGC YTA GTT GTT T	25
	PR	CTG TCC CGC CCA AGA TAC A	25
	Probe	FAM CCA GGA TTG GGT GGA ACC TGC AAA BHQ-1	5
RP	PF	CCA AGT GTG AGG GCT GAA AAG	15
	PR	TGT TGT GGC TGA TGA ACT ATA AAA GG	15
	Probe	FAM CCC CAG TCT CTG TCA GCA CTC CCT TC BHQ-1	5

Centers for Disease Control and Prevention. Modern Methods for Influenza and Subtyping, Atlanta Georgia. Association of Public Health Laboratories. 2004: 81–84 [13].

### Reaction controls and interpretation

DNA or RNA lyophilizates (AmpliRun® Vircell; Granada–Spain) were used as the positive controls for all evaluated viruses. All samples that presented amplification for any of the viral markers plus the internal control were considered positive results. All samples without amplification of the viral markers but with amplification of the internal control were considered negative results. Samples that did not present amplification of the internal control were considered inadequate.

### Statistical analysis

Descriptive statistics were used to analyse the prevalence of the viruses included in the study, with the percentages given with a 95% confidence interval. The Chi-square test of homogeneity and independence and Fisher's exact test were used to compare categorical variables (p-values <0.05 were considered significant). ANOVA and Student’s t-test were used for hypothesis testing of the quantitative variables. The analyses were performed with the SPSS Statistics version 24.0 software, and the graphics were generated with the Statgraphics^®^ Centurion XVI.II and Microsoft^®^ Excel^®^ 2010 software.

### Ethics statement

The information of the biological specimens used in the present study is not traceable to the patient data identity. All the samples were used in an anonymous way.

The study was approved by the Ethics Committee and the Research Committee of the National Committee of Scientific Research of the Instituto Mexicano del Seguro Social with the registration number R-2016-785-041.

## Results

### Demographic data

**Fig 1:**
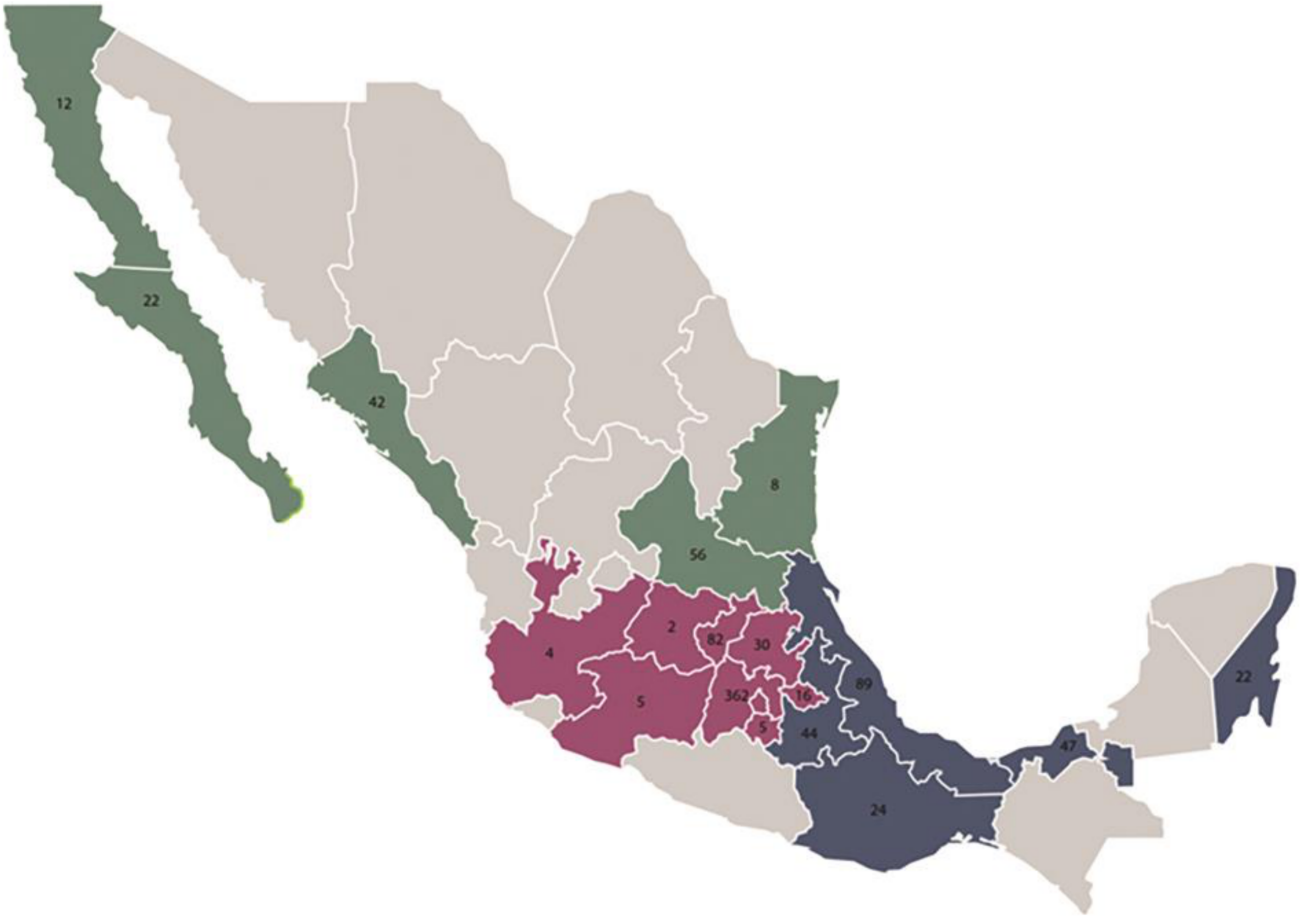
Demographic distribution of the analysed samples. In this figure, the distribution of the analysed samples is shown as follows: in green the north zone, in red the middle zone, and in blue the south zone.

Of the 872 samples analysed, 451 were from males (51.7%) and 421 were from females (48.3%). The average age was 41 years, with minimum and maximum extremes of 0 and 101 years, respectively. The population was divided into 4 age groups based on the age ranges included on the Instituto Mexicano del Seguro Social (IMSS; Mexican Social Security Institute) health cards. According to this stratification, the samples were distributed as follows: 265 samples corresponded to children 0–9 years of age (30.4%), 28 to young people 10–19 years of age (3.2%), 263 to adults 20–59 years of age (30.2%), and 316 to adults 60 years and older (36.2%). (Table 2). UnintentionallyA, the analysed samples were collected from patients from three geographical areas of the country as follows: the north zone (Baja California, Baja California Sur, Sonora, Chihuahua, Coahuila, Nuevo León, Durango, Sinaloa, Zacatecas, San Luis Potosí, Tamaulipas, Nayarit, and Aguascalientes) for 16.1% of the samples, the central zone (Guanajuato, Querétaro, Hidalgo, Morelos, Jalisco, Colima, Michoacán, Estado de México, Tlaxcala, and Distrito Federal [Mexico City]) for 58.0% of the samples, and the south zone (Puebla, Guerrero, Veracruz, Oaxaca, Tabasco, Chiapas, Campeche, Yucatán, and Quintana Roo) for 25.9% of the samples (Fig 1). Additionally, these data were analysed based on the age groups (S2 Table and S1 File).

**Table 2:** Demographic and clinical data of the analysed samples.

Sex	n	%
Male	451	51.7
Female	421	48.3
Age group (years)	n	%
0–9	265	30.4
10–19	28	3.2
20–59	263	30.2
≥ 60	316	36.2
Zone	n	%
North	140	16.1
Central	506	58.0
South	226	25.9
Clinical situation	n	%
Hospitalized	812	93.1
Ambulatory	60	6.9
Comorbidities	n	%
Total	348	39.9
Diabetes	189	21.7
Obesity	91	10.4
Smoking	70	8.0
Immunosuppression	58	6.7
Asthma	40	4.6
HIV	14	1.6
Pregnancy	10	1.1
Symptoms	n	%
Average number of symptoms	7.61	——
Cough	794	91.1
Dyspnoea	701	80.4
Fever	633	73.0
Headache	545	62.5
Rhinorrhea	500	57.3
Prostration	488	56.0
Myalgia	447	51.3
Chest pain	444	50.9
Chill	432	49.5
Arthralgias	386	44.3
Odinophalgias	372	42.7
Abdominal pain	204	23.4
Irritability	186	21.3
Conjunctivitis	165	18.9
Diarrhea	118	13.5
Cyanosis	111	12.7
Polypnea	111	12.7

### Prevalence and positivity

Of the total samples analysed, 312 were positive for at least 1 virus (35.8%), with 47.1% from female patients (n = 147) and 52.9% from male patients (n = 165); there was no significant difference between the groups (p>0.05). Of the total positive samples, RV had the highest number of detections (33.0%), followed by HRSV (30.8%), HMPV (10.6%), HMdV (9.6%), HPIV3 (8.7%), βCoV1 (8.7%), EV (5.8%), and PBpV (4.5%). The other viruses presented percentages below 4%, although together they accounted for 10.3% of all positive cases. Only HPIV2 was not detected (Table 3)

**Table 3:** Results of RT-qPCR test.

Overall results	Detection n/N (%)	IC 95%
Negative	560/872 (64.2)	61.0–67.4
Positive (at least 1 virus)	312/872 (35.8)	32.6–39.0
Virus	Detection n/N (%)	IC 95%
RV	103/312 (33.0)	27.8–38.2
HRSV	96/312 (30.8)	25.6–35.9
HMPV	33/312 (10.6)	7.2–14.0
HMdV	30/312 (9.6)	6.3–12.9
HPIV 3	27/312 (8.7)	5.5–11.8
βCoV1	27/312 (8.7)	5.5–11.8
EV	18/312 (5.8)	3.2–8.4
PBpV	14/312 (4.5)	2.2–6.8
HPIV 4	11/312 (3.5)	1.5–5.6
HCoV 229E	8/312 (2.6)	0.8–4.3
HPIV 1	7/312 (2.2)	0.6–3.9
HCoV NL63	4/312 (1.3)	0.03–2.5
HCoV HKU1	2/312 (0.6)	-0.24–1.5
HPIV 2	0/312 (0.0)	——

### Co-infections among respiratory viruses that cause ARIs

**Fig 2:**
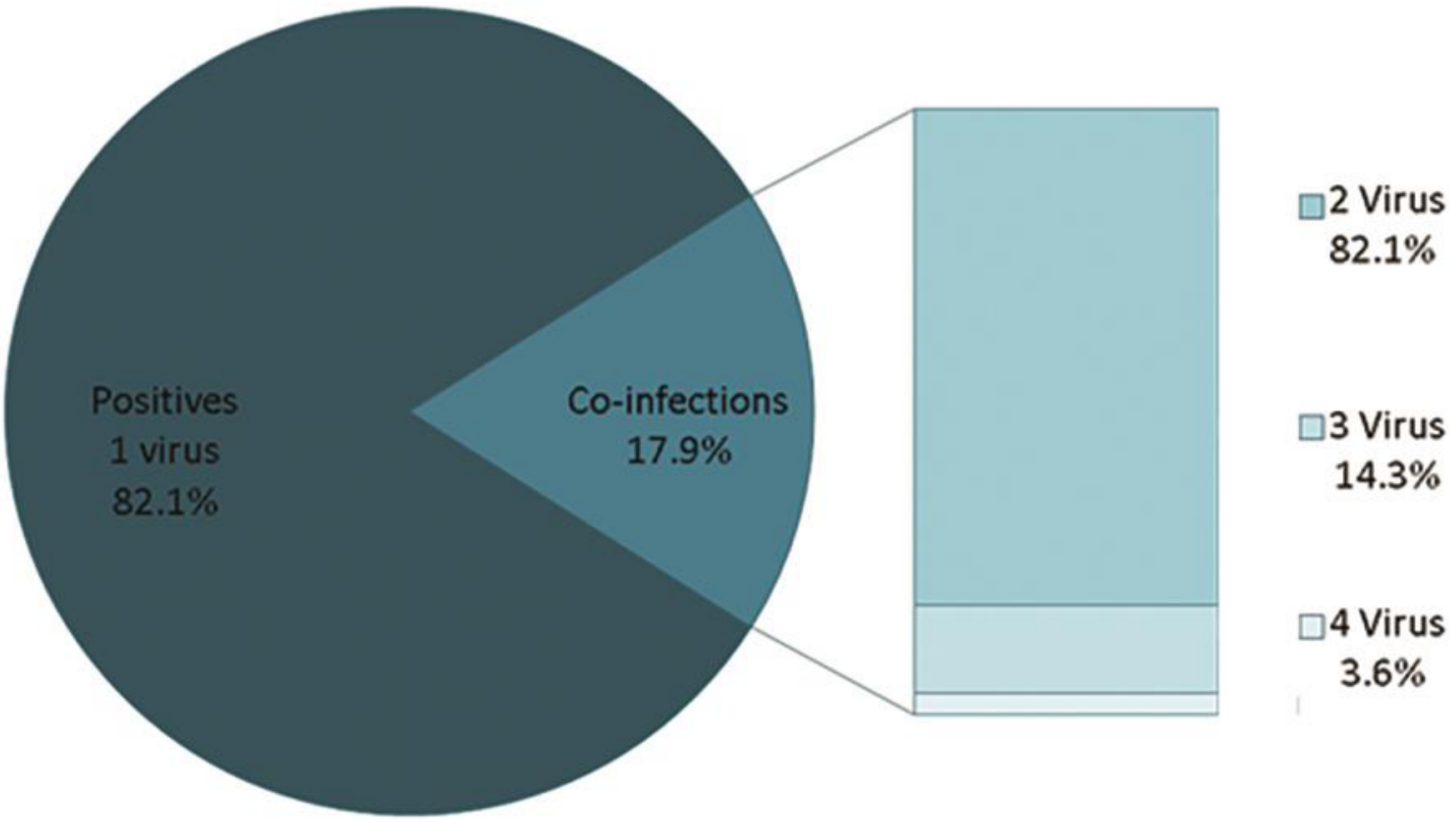
Virus co-infections in ARI cases. The figure shows the distribution of the co-infections, the circle represents the universe of positive samples (N = 312), whereas the number of viruses involved in the co-infections is broken down in the rectangle (N = 56).

A total of 56 cases of co-infection were identified, corresponding to 6.4% of the total analysed samples and 17.9% of the positive samples. Among the observed co-infections, 46 were caused by 2 viruses (82.1%), 8 by 3 viruses (14.3%), and 2 by 4 viruses (3.6%) (Fig 2).

The 3 viruses most often identified in cases of co-infection were RV in 48.3% of the cases, followed by HRSV (35.7%) and HMdV (32.1%). Interestingly, in the case of PBpV, despite it being the causal agent of only 19.6% of co-infections, these co-infections represented 78.6% of the PBpV cases detected. The behaviour of HMdV was similar, with 60% of all cases in which this virus was present involving a combination with other viruses. Both of these cases represent a significant association (p<0.05) with co-infection (Table 4).

**Table 4:** Viruses participation in co-infections.

Etiologic agent	Co-infection of 2 Viruses	Co-infection of 3 Viruses	Co-infection of 4 Viruses	Total	Percentage of total co-infections (N = 56)	Percentage of cases associated with co-infection. (%)
RV	21	6	0	27	48.2	26.2
HRSV	15	3	2	20	35.7	20.8
HMdV	13	3	2	18	32.1	60.0
βCoV1	9	2	2	13	23.2	48.1
HMPV	9	0	0	9	16.1	27.3
HPIV 3	7	3	0	10	17.9	37.0
PBpV	5	4	2	11	19.6	78.6
EV	4	2	0	6	10.7	33.3
HPIV 4	3	1	0	4	7.1	36.4
HPIV 1	2	0	0	2	3.6	28.6
HCoV 229E	1	0	0	1	1.8	12.5
HCoV HKU1	1	0	0	1	1.8	50.0
HCoV NL63	0	0	0	0	0.0	0.0
HPIV 2	0	0	0	0	0.0	0.0

The most commonly observed viral combination was HMdV + RV (16.1%), followed by HPIV3 + RV and HRSV + βCoV1 (both 7.1%). Although it occurred only 2 times, the co-infection with 4 viruses involved the same agents in both cases (HRSV + HMdV + βCoV1 + PBpV).

A comparison was performed between the days of hospitalization required by the patients with negative samples and those with samples positive for a single virus or in whom 2 or more viruses were detected. The analysis of this clinical data showed that the hospitalization was significantly higher in the negative samples than in the positive ones for a virus or with co-infections: 7.5, 5.1 and 4.5 days on average, respectively (p <0.05). We observed the same tendency with the average number of comorbidities and symptoms: 0.65 in the negative samples versus 0.35 in positive samples (p <0.05) and 7.7 in the negative samples against 7.4 in positive samples (p > 0.05), respectively. Nevertheless, the number of clinical symptoms and comorbidities were greater in the ARIs positive for a single virus than in the patients with co-infections (p <0.05) (Table 5).

**Table 5:** Hospitalization time.

	Positive	Negative	P-Value	Positive for 1 virus	Coinfections	P-Value
Days of hospitalization	5.1 ± 4.5	7.5 ± 9.1	*P<0.05	5.2 ± 4.5	4.5 ± 4.1	P>0.05
Number of clinical symptoms observed	7.4 ± 2.9	7.7 ± 2.8	P>0.05	7.6 ± 2.8	6.2 ± 2.8	*P<0.05
Comorbidities	0.35 ± 0.71	0.65 ± 0.78	*P<0.05	0.41 ± 0.76	0.07 ± 0.26	*P<0.05

### Distribution of cases by age group

**Fig 3:**
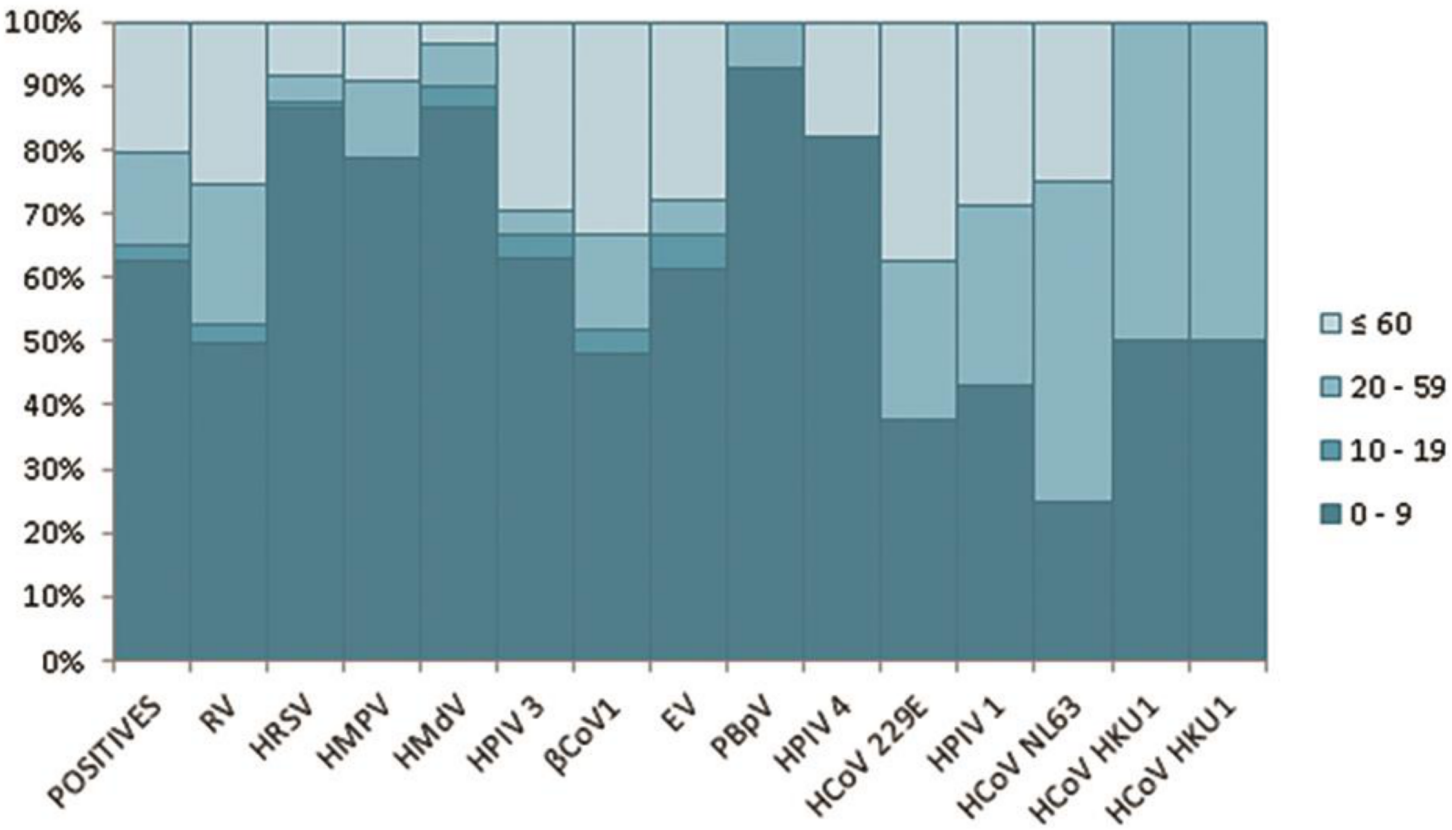
Proportion of positive cases by age group. The figure shows the detection percentage of each of the 13 non-influenza respiratory viruses identified in this study by age group.

The highest number of positive cases occurred in the group aged 0 to 9 years (62.5%) and the second highest in the group aged 60 years or older (20.5%), followed by the 20- to 59-year-old group (14.4%) and finally the 10- to 19-year-old age group (2.6%), which represented a significant difference (p<0.05). Consistently, the 0- to 9-year-old age group comprised 91.1% of the co-infections. Interestingly this group contained 92.9% of all PBpV infections, 86.7% of the HMdV infections, and 86.5% of the HRSV cases (Fig 3).

### Seasonality

**Fig 4:**
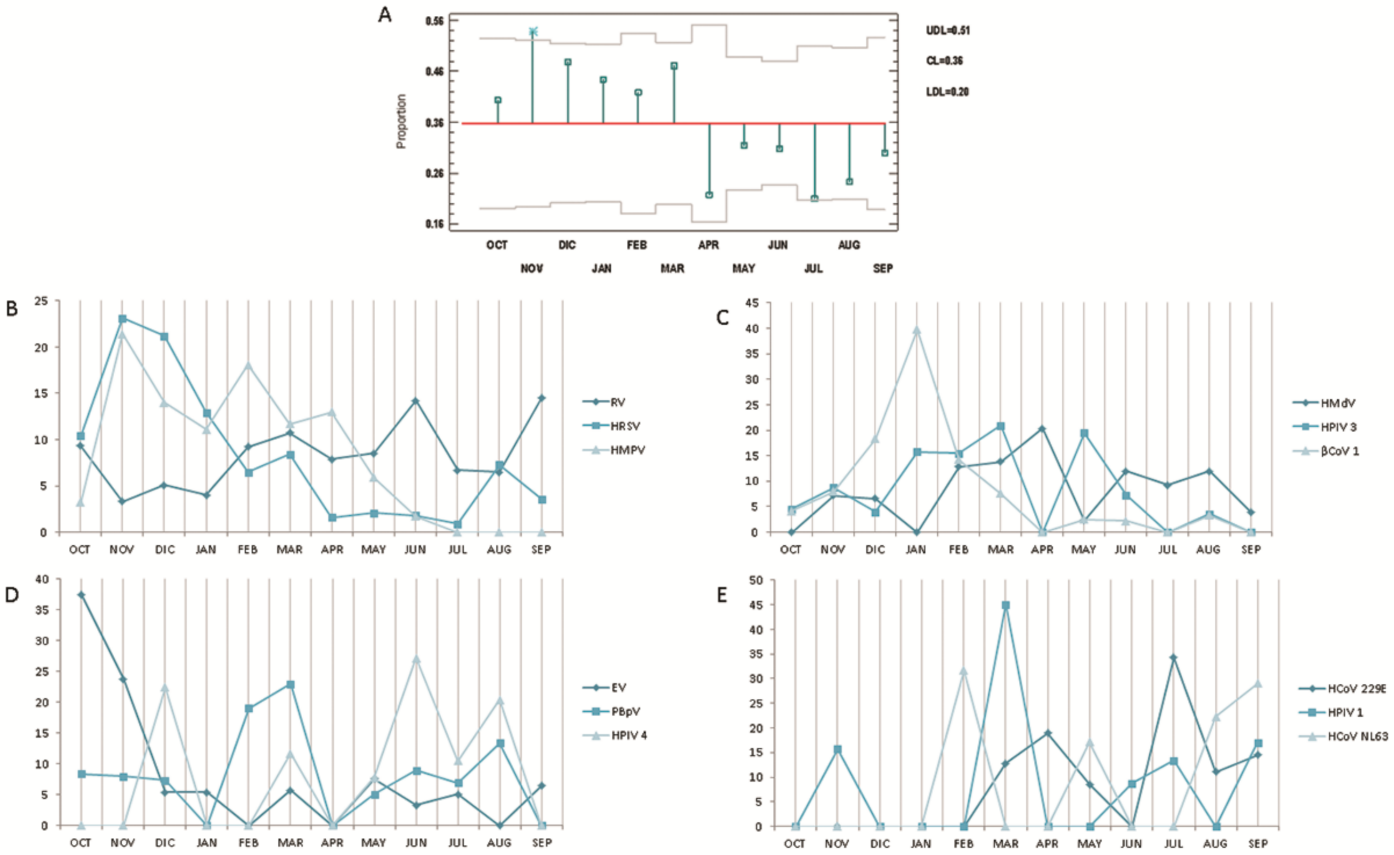
Seasonality of the non-influenza respiratory viruses. Seasonality of the non-influenza respiratory viruses. (A). Analysis of means (ANOM) where it was determined that the month of November, highlighted with an asterisk, had a ratio of viral detection significantly higher than other months, falling outside the decision limits (UDL = 0.51; CL = 0.36; LDL = 0.20). (B) Seasonality of RV, HRSV, and HMPV are shown; (C) Seasonality of HMdV, HPIV3, and βCoV1 are shown; (D) Seasonality of EV, PBpV, and HPIV4 are shown; (E) HCoV 229E, HPIV1, and HCoV NL63 are shown. In B, C, D and E, the percentages represent the distribution of the monthly prevalence of each virus. HPIV2 and HCoV HKU1 were not graphed due to the insufficient numbers of positive samples.

When conducting the analysis of the overall behaviour of the viruses in the period covered by the study, we observed that the months with high and low positivity differed significantly (p<0.05). Fig 4B–4E shows the monthly behaviour of each virus. The month of November presented the highest percentage of cases during the year **(**Fig 4A**)**.

When the analysis was performed by season, we observed differences between the four seasons of the year (p<0.05). Generally, summer had a significantly lower proportion of positive samples (24.5%; p<0.05), whereas fall had the highest proportion (44.9%; p<0.05).

However, the individual trends of the viruses were not the same. For instance, spring (March 21 to June 20) accounted for the greatest proportions of RV, HMdV, HPIV4, and HPIV1 cases (29.5%, 38.5%, 46.8%, and 48.0%, respectively). In contrast, most HRSV (48.2%) and EV (72.6%) cases were detected in the fall (September 21 to December 20), and winter (December 21 to March 20) presented the highest prevalence of HMPV (47.3%) and βCoV1 (60.5%). The other viruses (HCoV 229E, HCoV HKU1, HPIV 3, PBpV, and HCoV NL63) were detected throughout the year, with no seasonal trends observed (p>0.05).

## Discussion

Approximately 27 million ARI cases occur annually in Mexico [14]. These cases can be caused by a large variety of aetiological agents. However, the main purpose of epidemiological surveillance in the country is to detect the antigenic variations of influenza that are presented each season, which determine the changes in the vaccine composition. In 2013, the Instituto de Diagnóstico y Referencia Epidemiológicos (InDRE; Institute of Epidemiological Diagnosis and Reference) implemented a differential diagnosis of influenza that included 14 other respiratory viruses [15]. Due to this, at this moment Mexico does not have enough epidemiological information about the great diversity of viruses causing Acute Respiratory Infection. This lack of information, makes physicians search only for influenza, it is therefore the most requested confirmatory test in the laboratory during the whole year, even when we are out of influenza season (season comprises from October to May), leading to useless negative influenza results in most of the cases, without identifying the real causal agent of ARI in Mexico. This problem have direct implications for each patient, as the clinician does not know the causal agent, the patient does not receive the suitable treatment. It also has implications that affect the whole society, because the ignorance of the circulation patterns and incidence of other respiratory viruses limit preventive actions by health institutions.

According to data from the Secretary of Health, approximately 80% of the samples from patients with ARIs that are received for confirmation of influenza virus infection outside of flu season are negative for the different strains of this virus and remain without a defined aetiology. Therefore, the objective of this study was to determine the viral aetiology of these infections and to analyse the behaviour of non-influenza respiratory viruses in the Mexican population.

Beginning and ending with the 2015 influenza season, 872 samples collected over one year were evaluated to determine the presence of HMPV, HRSV, HPIV 1–4, βCoV1, HCoV, HMdV, RV, EV, and PBpV. These 14 respiratory viruses share symptoms with influenza but are rarely suspected or can be confused with influenza.

In contrast to other studies in other countries investigating this issue, this study included samples corresponding to all age groups and regions of the country. Therefore, the study population best represents the demographic distribution of ARIs in the country.

In contrast to most of the prevalence studies of ARIs, in which all age groups are not normally analysed and data is limited to a single region of the country, in this study, nor the age of the patient, nor the region of the country from which the sample came were included as inclusion or exclusion criteria. Therefore, the population analysed in this study represents better the demographic distribution of ARIs in our country.

This type of study helps provide relevant data for the development of treatment and prevention strategies because the currently available antiviral agents and vaccines are primarily directed at influenza infection. However, the proportion of ARIs caused by different influenza viral agents is not negligible, with the detection range of non-influenza respiratory viruses spanning from 16.5 to 72.7% in studies conducted worldwide [4;16–20], depending on factors such as the study design, study population, detection technique used, and period covered by the study. In our case, the prevalence of the analysed viruses was 35.8%, which was within the cited range. The most prevalent viruses were RV, HRSV, and HMPV, and the only virus that was not detected was HPIV2. This result was consistent with other studies in which type 2 parainfluenza virus had the lowest detection frequency [21–24]. However, there is evidence that its prevalence increases when HPIV-3 and HPIV-1 are reduced [25–26].

Consistent with our results, RV was identified as the most common virus in ARI cases in several studies [17;20;22;27] in which the presence of influenza and other respiratory viruses from throat swabs is determined by molecular techniques of own design or using commercial panels. Equal data were observed in a study of Mexican children [28]. This virus is the major aetiological agent of the common cold [29], however, it may also be involved in serious ARIs [30–32], which are primarily observed in patients with underlying comorbidities, such as asthma [33–35] or other chronic pulmonary diseases [36], indicating a certain degree of opportunism.

Similar to RV, HRSV was identified in high proportions in other studies [4;37], conducted in Poland in which 380 individuals were analysed without age selection, and in Mexico in which 383 children up to 5 years of age were studied. This trend is also observed in studies where a higher prevalence of influenza was observed [19;21;23;38], and HRSV has been associated with the pathogenesis of asthma. More importantly, HRSV is the leading cause of child mortality caused by viruses [39].

The third most prevalent virus was HMPV. In Mexico, the first study in which the presence of this virus was determined was published in 2005 [40]. Subsequently, other Mexican studies demonstrated its importance in the child population [16;41–42]. Studies such as that of Diaz et al. [16] demonstrated that HMPV was mostly associated with severe acute respiratory infections instead of mild and moderate infections.

The importance of the differential diagnosis of other respiratory viruses in samples with negative influenza results becomes apparent when we observe the prevalence of the three main viruses identified in this study as well as their associations with severe cases and deaths, especially in the child population.

Co-infections represented 17.9% of the positive samples. This percentage ranged between 6.9 and 18.6% in other studies that also included various age groups [20;43–44]. However, much higher percentages have been found (above 30%) in study populations composed of children under 5 years of age [16;22–23;45].

Our results confirmed that viral co-infection was common, especially in the child population because 91.1% of all co-infections occurred in the 0 to 9 year age group. This result could be attributed to slower viral elimination due to a still-developing immune system [46].

The virus most involved in co-infections was PBpV, as in 78.6% of the cases in which it was identified, it was found together with another virus. Studies have reported identification percentages of this virus in conjunction with other agents ranging from 47.4 up to 90% [47–48]. However, its prevalence in asymptomatic patients is also high (44% or 43%) [49], which calls into question whether PBpV by itself is capable of generating disease [50] or if it only participates as a facilitator for another agent to infect the host. At present, cellular and animal models are still being developed for this virus [51], therefore, the evidence needed to confirm this hypothesis does not yet exist.

From a clinical perspective, whether the presence of a co-infection results in a more serious case or is a poor prognostic factor is a matter of controversy within the scientific community. Although some studies have reported cases with these characteristics [52–54], reports by other authors have suggested that co-infections are not synonymous with clinical differences or greater severity [55–56].

In the study of Martínez-Roig et al. [57], an inverse relationship was found between the number of viruses detected and the hospitalization time as well as the need for oxygen therapy. A similar relationship was observed in the study of Canducci et al. [58], where there was a greater hypoxia and lengthier hospitalization time for infections caused purely by HRSV compared to coinfections. In our study, the ARIs caused by a single virus also presented lengthier hospitalization times than ARIs caused by 2 or more viruses, although the differences were not statistically significant.

Notably, similar to the estimation of prevalence, differences in the reported ranges of and discrepancies in the severity associated with viral co-infections can be attributed to the detection techniques employed, the population, the time period of the study, and the viruses studied.

Because of the short period of time comprised by this study and the limited number of samples, it is not possible to state, but it can be suggested certain seasonal behaviors of some studied viruses. Generally, the peak of respiratory infections occurs in the period between November and April in countries of the northern hemisphere [59–60]. The influenza season is well known in Mexico and worldwide; however, the seasons of other respiratory viruses are not well known. According to our results, the highest prevalence of these viruses in Mexico appears to occur from October to March (autumn and winter), which coincides with the influenza season. Detection was significantly higher in November of 2014; during this month, there was a marked decrease in the national mean temperature from 23.3 to 18.3°C. According to Cui et al. [61], mean temperature is the key climatic parameter associated with the prevalence of many respiratory viruses because some viruses survive and/or replicate better at low temperatures [61]. On the other hand, Viegas et al [62] proposed that mucus release by cilia was reduced when the temperature of the respiratory tract was lowered; consequently, the local innate immune response (neutrophil and natural killer (NK) cell migration) was also reduced, thereby promoting viral infection.

Climatic factors may also indirectly favour the transmission efficiency because low temperatures induce a change in social behaviour that favours interior overcrowding and increases the likelihood of close contact and transmission of infections [63]. For this reason, it is a common suggestion to avoid going to school or work when you are undergoing an infectious process.

Our results suggest that some viruses have a more marked seasonal trend than other viruses. In winter, the prevalences of HMPV, HPIV3, and BCoV1 were higher than in the other seasons of the year. The circulation of HMPV in particular predominates in the colder seasons because a marked reduction in its prevalence is observed in the spring until it disappears completely in the summer. Furthermore, in a study conducted in Mexico City, in which 525 children between 1 and 15 years of age were analysed and in other study performed in China with 607 hospitalized patients was reported that the highest prevalence of HMPV occurs in the winter and that its seasonality overlaps with that of other viruses [19;43].

Similar to HMPV, some authors agree that HRSV is a virus that circulates preferentially in colder seasons [19;43;64]. This study demonstrates that HRSV can cause ARIs in all seasons of the year but is consistent with other studies that report that its prevalence is highest during the fall and winter.

Conversely, RV seemed to be the virus best suited to the climatic conditions of the country because there were no significant differences in its prevalence in the different seasons. According to the results of a study conducted in China [61], the optimal circulation temperature range of this virus is 15 to 25°C, which is similar to the range of the mean Mexican temperature and may justify its high prevalence in all months of the year.

However, despite all of the information reported to date, factors affecting the circulation of different viruses remain unclear. Annual research is needed to establish the seasonality of these viruses with more accuracy and precision.

## Conclusion

Although influenza is one of the main causative agents of respiratory infections worldwide, it is of vital importance to determine the prevalence and timing of other causal agents. In this study, we identified 13 non-influenza respiratory viruses that occurred in any season of the year. This study provides evidence for the prevalence and seasonality of a wide range of respiratory viruses circulating in Mexico that constitute a risk for the population. Additionally, our data suggest that including these tests more widely in the diagnostic algorithm for influenza could reduce the use of unnecessary antibiotics, reduce the hospitalisation time, and enrich national epidemiological data regarding infections caused by these viruses.

## Supporting information

S1 Table Performed tests. (DOCX)

S2 Table Demographic and clinical data of analysed samples by age group. (DOCX)

S1 File Prevalence of non-influenza data. (XLSX)
