# Supplementary material for: Prevalence of non-influenza respiratory viruses in acute respiratory infection cases in Mexico
Source: PLoS One. 2017 May 3;12(5):e0176298. doi: 10.1371/journal.pone.0176298 (PMC5415110; doi:10.1371/journal.pone.0176298)
Supplement: S1 Table — (DOCX) [file pone.0176298.s001.docx]

|  | Sample Selection | Study |
| --- | --- | --- |
|  | Influenza viruses | Non- Influenza viruses |
|  | Influenza A H1N1 pdm, Influenza A H1N1,  Influenza A H3N2,  Influenza B. | RV, HRSV, HMdV, BCoV1, HMPV, HPIV 3, PBpV, EV, HPIV 4, HPIV 1, HCoV 229E, HCoV HKU1, HCoV NL63 and HPIV 2. |
| Samples | Not apply | 872 |
