# Supplementary material for: Prevalence of non-influenza respiratory viruses in acute respiratory infection cases in Mexico
Source: PLoS One. 2017 May 3;12(5):e0176298. doi: 10.1371/journal.pone.0176298 (PMC5415110; doi:10.1371/journal.pone.0176298)
Supplement: S2 Table — (DOCX) [file pone.0176298.s002.docx]

| Age group (years) | n (%) | Male  n (%) | Female  n (%) | North  n (%) | Central  n (%) | South  n (%) | Hospitalized n (%) | Ambulatory n (%) | Comorbidities n (%) | Number of symptoms (average) |
| --- | --- | --- | --- | --- | --- | --- | --- | --- | --- | --- |
| 0 - 9 | 265 (30.4) | 154 (58.1) | 111 (41.9) | 47 (17.7) | 130 (49.0) | 88 (33.2) | 253 (95.5) | 12 (4.5) | 25 (9.4) | 7.1 |
| 10 - 19 | 28 (3.2) | 10 (35.7) | 18 (64.3) | 6 (21.4) | 17 (60.7) | 5 (17.9) | 24 (85.7) | 4 (14.3) | 8 (28.6) | 7.1 |
| 20 - 59 | 263 (30.2) | 127 (48.3) | 136 (51.7) | 35 (13.3) | 161 (61.2) | 67 (25.5) | 227 (86.3) | 36 (13.7) | 136 (51.7) | 8.1 |
| ≥ 60 | 316 (36.2) | 160 (50.6) | 156 (49.4) | 52 (16.4) | 198 (62.6) | 66 (21.0) | 308 (97.5) | 8 (2.5) | 179 (56.6) | 7.6 |
